# The impact of functional correlations on task information coding

**DOI:** 10.1162/netn_a_00402

**Published:** 2024-12-10

**Authors:** Takuya Ito, John D. Murray

**Affiliations:** Department of Psychiatry, Yale School of Medicine, New Haven, CT, USA; Thomas J. Watson Research Center, IBM Research, Yorktown Heights, NY, USA; Department of Neuroscience, Yale School of Medicine, New Haven, CT, USA; Department of Physics, Yale University, New Haven, CT, USA; Department of Psychological and Brain Sciences, Dartmouth College, Hanover, NH, USA

**Keywords:** Neural coding, Functional connectivity, fMRI, Noise correlations, Signal correlations, MVPA, Multitask

## Abstract

State-dependent neural correlations can be understood from a neural coding framework. Noise correlations—trial-to-trial or moment-to-moment covariability—can be interpreted only if the underlying signal correlation—similarity of task selectivity between pairs of neural units—is known. Despite many investigations in local spiking circuits, it remains unclear how this coding framework applies to large-scale brain networks. Here, we investigated relationships between large-scale noise correlations and signal correlations in a multitask human fMRI dataset. We found that task-state noise correlation changes (e.g., functional connectivity) did not typically change in the same direction as their underlying signal correlation (e.g., tuning similarity of two regions). Crucially, noise correlations that changed in the opposite direction as their signal correlation (i.e., anti-aligned correlations) improved information coding of these brain regions. In contrast, noise correlations that changed in the same direction (aligned noise correlations) as their signal correlation did not. Interestingly, these aligned noise correlations were primarily correlation increases, suggesting that most functional correlation increases across fMRI networks actually degrade information coding. These findings illustrate that state-dependent noise correlations shape information coding of functional brain networks, with interpretation of correlation changes requiring knowledge of underlying signal correlations.

## Author Summary

We leveraged insights from the neural coding theory to interpret large-scale patterns of correlated activity across many cognitive tasks in human fMRI. We characterized the signal correlation (i.e., task tuning similarity between regions), the noise correlation (i.e., functional connectivity), and the change in noise correlation by task engagement. Following neural coding theory, we parsed noise correlation changes into components that are aligned with the signal correlation, which degrade information coding across regions, versus those that are anti-aligned, which enhance information coding. Anti-alignment of noise correlation changes with the correlation minimizes the amount of interference between the two brain regions. Together, these findings provide a task information coding perspective to interpret task-state correlation changes in human functional brain networks.

## INTRODUCTION

Advances in functional brain imaging have enabled the investigation of the large-scale network organization of the human brain. In resting-state functional magnetic resonance imaging (fMRI), studies have found highly reliable and modular functional connectivity (FC) organization, which is measured through correlating the spontaneous fMRI activity of different brain regions (Power et al., 2011; Yeo et al., 2011). Related studies have shown that this overall network organization persists across task states (Cole, Bassett, Power, Braver, & Petersen, 2014; Gonzalez-Castillo & Bandettini, 2018; Krienen, Yeo, & Buckner, 2014), disease states (Spronk et al., 2021), and individuals (Gratton et al., 2018). Despite the appearance of state- and trait-invariant network organization, there are reliable changes that occur to the network organization for specific networks or regions (Cole et al., 2014; Krienen et al., 2014; Shine et al., 2016). While some recent methodological efforts in human brain imaging have worked to disambiguate the sources of state-specific network correlation changes (Cole, Yang, Murray, Repovš, & Anticevic, 2016; Duff, Makin, Cottaar, Smith, & Woolrich, 2018), the significance and interpretation of these correlation changes remain unclear.

**Figure 1.**
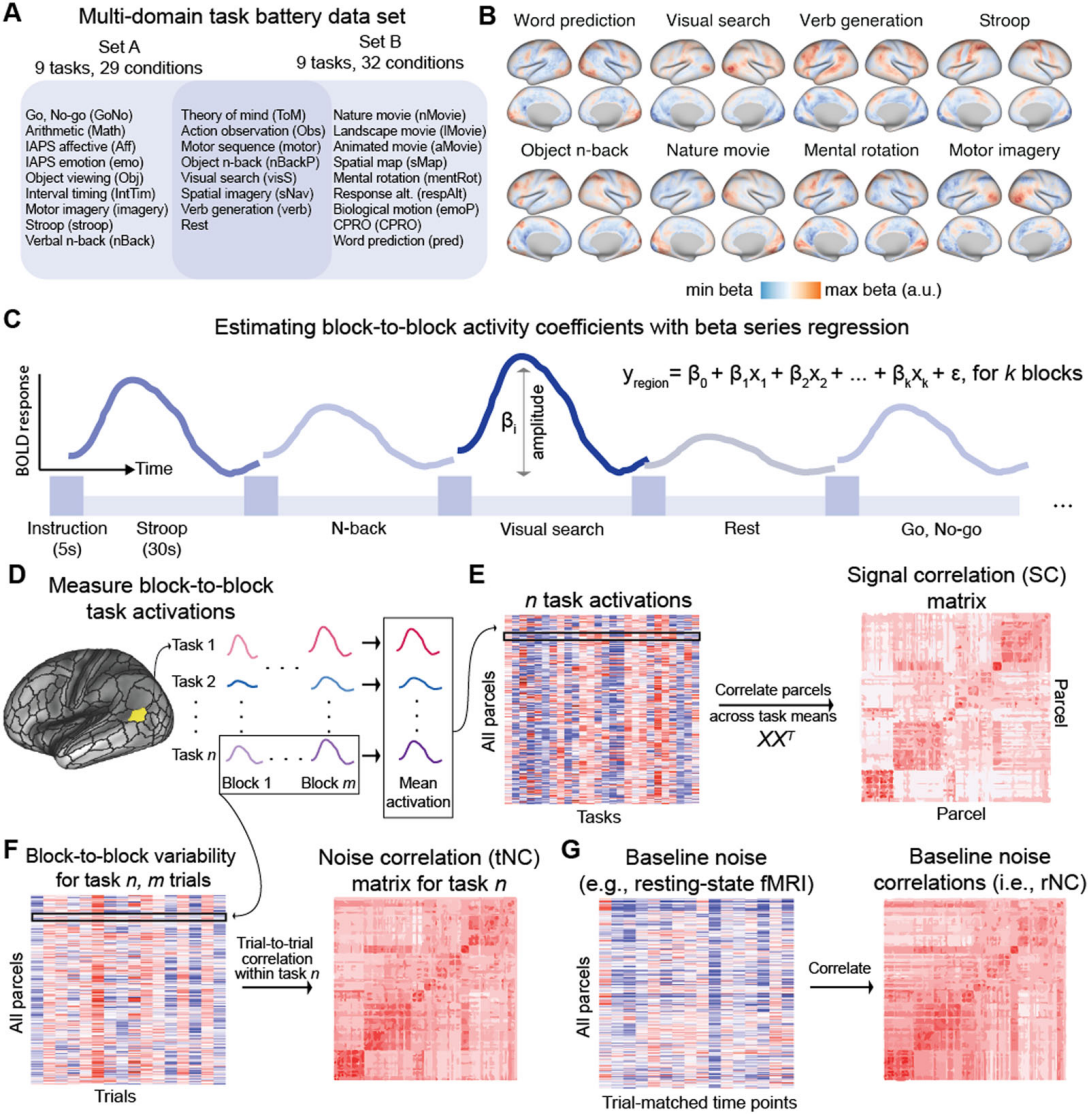
We used a multitask dataset to capture the large-scale SC and NC organization in human functional brain networks. (A) The MDTB dataset with 26 unique tasks (King et al., 2019). (B) Cortical activation maps for eight example tasks. (C) Blockwise activation estimates were obtained using a beta series regression approach, where each task block was modeled independently in a linear regression model (Rissman et al., 2004). (D) SCs and NCs in large-scale fMRI data are estimated from orthogonal timeseries sources. We estimate the trial-to-trial task activation amplitude in fMRI data for each region and for all tasks. (E) To estimate the SC matrix, we compute the correlation between all pairs of brain parcels using the cross-trial mean activation of many tasks. (F) In contrast, tNC matrices for a given task are computed as the correlation of trial-to-trial variability between pairs of parcels within a single task. (G) The tNC can be compared with the well-studied baseline rNC using the resting-state fMRI activity. SCs and NCs are computed for each participant separately and then averaged to produce a group-level matrix estimate.

In parallel, empirical and theoretical neurophysiological studies have established a rigorous statistical framework to study the properties of neural correlations and how they impact neural coding (da Silveira & Berry, 2014; Moreno-Bote et al., 2014; Panzeri, Moroni, Safaai, & Harvey, 2022). Critically, there are two forms of correlated activity that contain distinct sources of variance within neural data, yet provide complementary information about task coding: the signal correlation (SC) and the noise correlation (NC; Cohen & Kohn, 2011). Intuitively, SC measures the tuning similarity between a pair of neural units. NC measures the temporal correlation during a task/stimulus (e.g., timepoints or trials; Figure 1C), capturing the dynamic interaction of two units. (Note that the terms SC and NC were originally defined through the lens of information theory, where “signal” corresponds to the mean across responses, and “noise” corresponds to the variance across responses [MacKay, 2003]. In the context of prior fMRI literature, these SCs and NCs are statistically equivalent to across-task coactivations and FC, respectively [Cole et al., 2019].) Under the neural coding framework, studies have suggested that the effect an NC has on task coding depends on how well it aligns (i.e., shares the same sign) with the underlying SC of those two units (da Silveira & Berry, 2014; Moreno-Bote et al., 2014; Panzeri et al., 2022). In particular, the signal-noise angle—the difference in the directions (sign and magnitude) of the SC and NC—determines the information coding properties of a neural population (Panzeri et al., 2022). This is because if an NC aligns with its SC, this would interfere with the coding direction of these two units. While the theoretical account of SC/NC was developed to account for empirical phenomena observed at the level of neuron pairs during the presentation of fine-grained sensory stimuli (Cohen & Kohn, 2011; da Silveira & Berry, 2014; Moreno-Bote et al., 2014) and local fMRI voxels (Cheng, Zhang, & Zhang, 2022; van Bergen & Jehee, 2018; Zhang, Wei, & Kay, 2020), the statistical principles are generic to account for neural data across a wide range of spatial and cognitive scales, including large-scale brain networks. Thus, we sought to investigate whether the SC/NC coding principles apply to the level of large-scale brain regions. A successful demonstration of SC/NC coding principles at the level of human functional brain networks would bridge the vast literature of FC analyses prevalent in the human functional connectomics literature with the rich neural coding framework developed in theoretical neuroscience.

SCs capture the similarity of task tuning between two neural units (neurons or brain regions). At the level of single neurons, this typically captures the tuning curve similarity of fine-grained sensory stimuli, such as the orientation of visual gratings (Cohen & Maunsell, 2009). While the impact of these correlations on task coding have been widely investigated in local spiking circuits, it remains unclear how this coding framework applies to large-scale brain networks. This is largely due to the fact that the types of fine-grained tuning curves (i.e., orientation gratings) captured in prior neurophysiology studies are generally inaccessible at the level of large-scale fMRI brain networks. Instead, large-scale fMRI brain networks have been previously shown to be selective to broader cognitive tuning curves, such as different cognitive tasks (Smith et al., 2009; Yeo et al., 2015). Thus, we leverage a multitask dataset that spans diverse cognitive domains to characterize the SC and NC organization across distributed functional brain networks. This approach plays to the strengths of fMRI, while allowing us to extend the prior theoretical neural coding framework to large-scale functional brain networks.

Here, we characterize the organization of the SC and NC in large-scale human brain networks and assess their coding properties across a wide range of cognitive tasks. While prior human neuroimaging studies primarily viewed correlations (i.e., FC) through the lens of dynamic communication (for a review, see Gonzalez-Castillo & Bandettini, 2018), we test whether FC can be interpreted through the lens of information coding. (Since FC and NC are statistically equivalent, we use them interchangeably in this study; see Table 1.) First, we extend the notion of SC from the correlation of visual tuning curves to a wide variety of cognitive tasks (i.e., *cognitive tuning curves*). We compared the organization of the SC to the well-established resting-state NC (rNC) organization of the human cortex, finding that the SC reflected a more modular and segregated network organization than rNC. Next, we built a statistical model of NC that demonstrated that, under the assumption that observed NC is driven by a linear combination of internal neural and external task sources, NCs should exclusively change in the direction of their underlying SC (i.e., positive increases in NC should be observed when the SC is positive). In contrast to this assumption, we found that NC changes do not typically align with the underlying SC in empirical fMRI data. Instead, a majority of NCs changed in the direction that was opposite to the SC. To understand the functional relevance of these NC changes, we leveraged the hypothesis from theoretical neuroscience that the alignment of SCs and NCs impacts the fidelity of task information coding. Indeed, we found that the signal-noise relationship is predictive of the fidelity of task coding in large-scale brain networks. These results shed light on the relationship between neural correlations and information coding, placing fMRI functional connectomics within the broader neural coding framework.

**Table 1.** Table of definitions and abbreviations

**Name**	**Abbreviation**	**Interpretation**
Signal correlation	SC	Similarity of task selectivity
Cross-task coactivations	n/a	*Across-task correlation of mean activations*
Noise correlation	NC	Neural/dynamic interactions
Functional connectivity	FC	*Correlation of ongoing activity*
Resting-state NCs	rNC	Spontaneous interactions/correlations
Resting-state FC	rFC	*Correlation of ongoing spontaneous variance*
Task-state NCs	tNC	Task-state interactions/correlations
Task-state FC	tFC	*Correlation of ongoing task-driven variance*
NC change	ΔNC	Task versus rest/baseline correlation change
Task vs. rest FC change	ΔFC	*Difference between task and rest correlations*

*Note*. Distinct terms used in different subfields within neuroscience are often computed identically and have converging interpretations. For example, NCs and FC are computed in a statistically identical manner and aim to capture a similar empirical phenomenon: interaction of neural units.

## RESULTS

### Estimating Multitask SCs and NCs in Human Functional Brain Networks

We first characterized multitask SC and NC across all pairs of parcels (see Table 1 for definitions). We used the publicly available multidomain task battery dataset collected by King and colleagues (King et al., 2019). Briefly, the multidomain task battery dataset contains 26 cognitive tasks per participant (Figure 1A). Tasks were interleaved across blocks, where each block was preceded by a 5 s instruction screen, followed by a 30-s block. For our analyses, we modeled the mean activity of each block separately for every brain region (parcel) in the Glasser atlas (Glasser et al., 2016) using a beta series regression (Rissman et al., 2004; Figure 1C).

To compute the SC between all pairs of parcels, we first computed the mean activation across blocks for each task separately (Figure 1D). This yielded a 360 parcel × 26 task matrix, from which we computed the SC matrix (Figure 1E). NC was calculated using the cross-block variability for every parcel, which is a distinct statistical property to the cross-block mean. (Note every task had the same number of blocks.) We calculated the NC between all pairs of brain regions and across all tasks (Figure 1F). To get a task-state NC (tNC) matrix, we averaged the NC across all tasks (excluding the resting-state condition). Resting-state blocks were also interleaved throughout the experimental design. To maintain consistency with how rNC and tNC were computed, rNC was computed in an identical manner to tNC (i.e., using a beta series regression; Figure 1G). Note that the across-block rNC matrix estimated here is quantitatively similar to the more common rNC that is computed across timepoints in the human neuroimaging literature (Supporting Information Figure S1). Conceptually, the approaches are equivalent in that NCs capture the variability across task responses, and SCs capture the mean across task responses. Here, we opt for a cross-block analysis, since it enables the characterization of task coding for each block, rather than across timepoints. There were no statistically significant differences in movement (i.e., framewise displacement) during rest and task blocks (Supporting Information Figure S2). Furthermore, we ensured both SC, rNC, and tNC matrices were highly reliable by measuring the reliability across subject splits, with a reliability of 0.81 or greater (Supporting Information Figure S3E).

### SCs Reveal a Highly Modular and Segregated Network Organization

**Figure 2.**
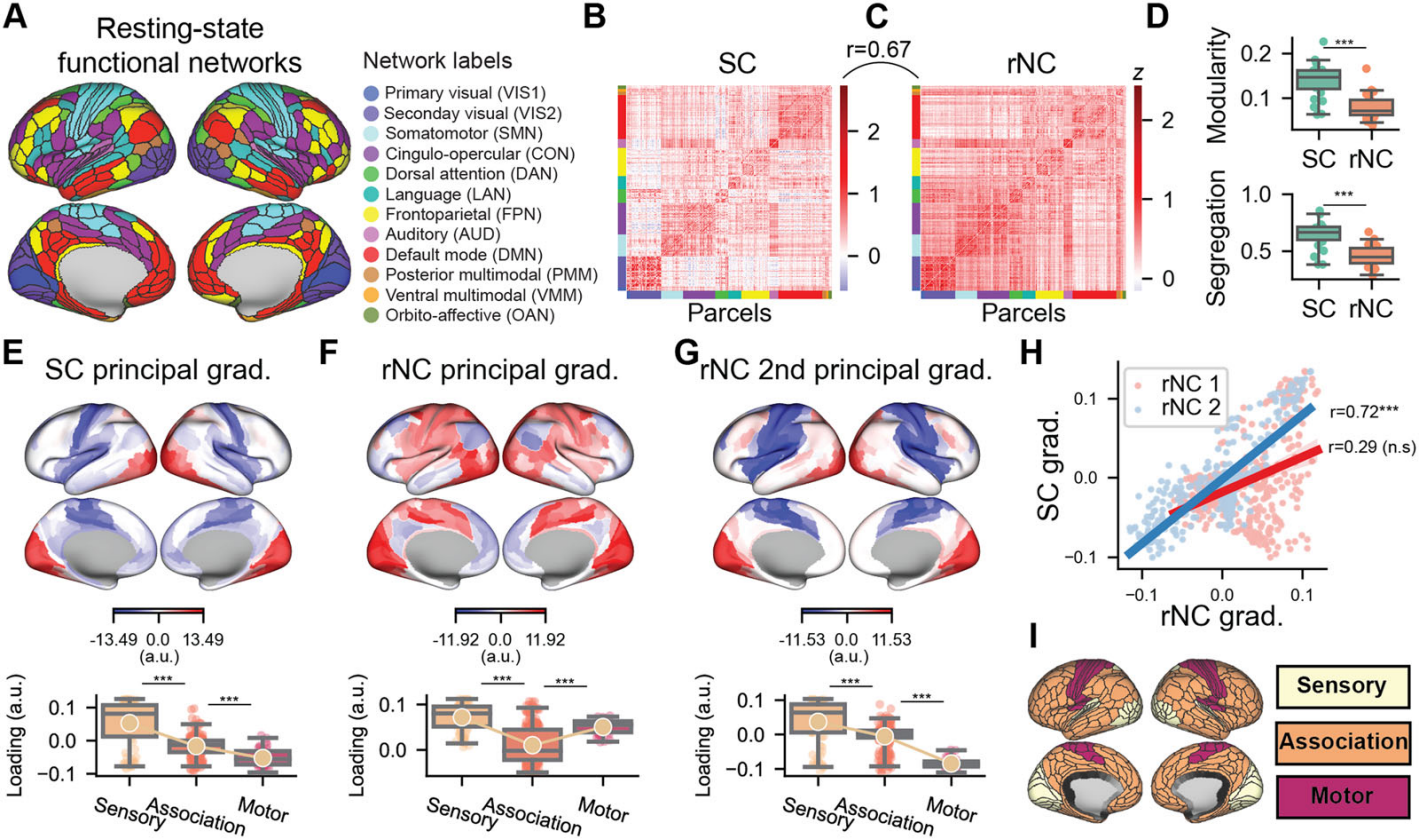
Comparing the SC matrix to the well-studied rNC matrix. (A) We used the Glasser parcellation with 360 cortical parcels. Parcels were partitioned into 12 functional networks (Ji et al., 2019). (B) The SC matrix, which captures the task-tuning similarity between pairs of brain regions. (C) The rNC matrix. (D) Modularity and segregation (using the Ji et al. partition) of the SC and rNC matrices. (E) Top: The first principal component of the SC matrix aligns along a sensory-association-motor gradient. Bottom: average loading projected onto three cortical systems. (F) The first principal component of the rNC is organized along the unimodal-transmodal (i.e., sensorimotor-association) hierarchy. (G) The second principal component of the rNC matrix also aligns along a sensory-to-motor gradient, and is (H) highly correlated with the SC principal gradient. (I) Sensory, association, and motor systems projected onto the cortex. A full comparison of the first three gradients of the SC and rNC can be found in Supporting Information Figure S4. Box plot bounds define the first and third quartiles (across participants), box whiskers indicate the 95% confidence interval, and the center line indicates the median.

We characterized the SC matrix in the context of the well-known rNC matrix. Prior work in rNC studies revealed a modular organization of functional brain networks (Figures 2A and 2C; Ji et al., 2019; Power et al., 2011; Yeo et al., 2011). These functional network divisions were identified using clustering and community detection algorithms on rNC matrices. To evaluate how the SC was related to this modular network organization, we computed the modularity and segregation of the SC with respect to the previously defined resting-state network partitions (Figure 2A). Modularity and segregation are related statistics that measure the strength of nodes within a network relative to the between-network connection strength (see the Methods section). Surprisingly, while the network partitions were optimized to maximize the modularity from rNC data, we found that the SC had both higher modularity and segregation than rNC (Figure 2C). This suggests that the SC can recapitulate the well-known functional subdivisions of cortex that are extracted from rNC.

Note, however, that empirically estimating the SC is a challenging problem. This is because when estimating within-subject SC, undesirable noise sources that are idiosyncratic with that individual and/or fMRI session may seep into the analysis, potentially confounding the “signal” with a subject-specific noise. One alternative to the naive approach of estimating SC within an individual is to compute the intersubject SC (Kim, Kay, Shulman, & Corbetta, 2018). This estimates the group-level SC using the task signal vector of one subject, with the task signal vector of another subject (or group of subjects). This isolates the task-driven signals, while ensuring that the individual-specific noise does not contaminate the SC. When computing the intersubject SC, we found strong correspondence with within-subject SC and intersubject SC (*r* = 0.81), providing validation that the SC provides reliable information about large-scale cognitive tuning curves (Supporting Information Figure S3).

### Cortical SCs Are Organized Along a Gradient of Functional Specialization

Complementing network analyses of the SC and NC, gradient analysis offers a way to capture the greatest axes of variation of the entire SC and NC matrices (Huntenburg, Bazin, & Margulies, 2018; Margulies et al., 2016). Gradient organization is computed by performing dimensionality reduction on the SC (or NC) matrices (e.g., a principal component analysis) and is complementary to network partitions as they exhibit smooth loadings/partitions, rather than “hard” or nonoverlapping networks (Huntenburg et al., 2018). The first gradient of the rNC matrix, which is equivalent to its first principal component, is the well-documented sensorimotor-association (or unimodal-transmodal) hierarchy that was first described by Mesulam (1998) and was subsequently identified in fMRI data (Margulies et al., 2016; Figure 2F). This unimodal-transmodal gradient is related to both transcriptomic variation (Burt et al., 2018) and myelination content, which is captured in the T1w/T2w contrast map (Glasser & Van Essen, 2011; 26.1% variance explained; Supporting Information Figure S4H). However, the gradient analysis of the SC matrix revealed a gradient of functional specialization, from sensory-association-motor areas (23.0% variance explained; Figure 2E; see Supporting Information Figure S4 for additional details.). Critically, when grouping together cortical systems into sensory, association, and motor systems (Figure 2I)—systems that are functionally distinct/specialized from each other—we found a monotonic relationship between these systems and their gradient loading. This is consistent with a gradient of functional specialization, where sensory and motor regions are defined by distinct functions, while association regions integrate the two (Ito & Murray, 2023). Moreover, this sensory-to-motor SC gradient was significantly associated with the second principal gradient of rNC (rank *r* = 0.72, nonparametric *p* < 0.001; Figure 2H). By comparison, the SC gradient was not correlated with the typical unimodal-transmodal gradient (i.e., the first rNC principal gradient; rank *r* = 0.29, *p* = 0.16). Note that the relative difference in variance explained between the first and second gradients was larger for the SC matrix (Δ3.68% for the rNC matrix and Δ5.45% in the SC matrix; see Supporting Information Figure S4H, suggesting that the re-ordering of the gradients in the SC matrix is not due to marginal changes in the amount of variance explained per gradient). Together, these results illustrate that while the SC preserves the overall functional brain network organization, it reveals a more cognitively specialized organization that more clearly delineates functionally specialized regions. This is consistent with the notion that SCs capture task selectivity similarities between brain regions.

### A Linear Model of State-Specific SC and NC Changes

A brain region’s functional specificity emerges from its pattern of connectivity, that is, its connectivity fingerprint (Mars, Passingham, & Jbabdi, 2018; Passingham, Stephen, & Kötter, 2002). Thus, two regions with similar functions or tuning curves (i.e., high positive SC) are likely to have high amounts of shared spontaneous activity (due to strong functional connections; i.e., high positive rNC). We verified this in our empirical data, finding that the SC matrix had overall strong correspondence with the rNC (rank *r* = 0.67, *p* < 0.0001; Figure 2B–C). However, how should stimulus-driven activity interact with spontaneous activity? To gain intuition on the interaction between stimulus-driven and spontaneous activity, we constructed a statistical model to simulate how state-specific NC emerges from an anatomically constrained network model with linear dynamics.

**Figure 3.**
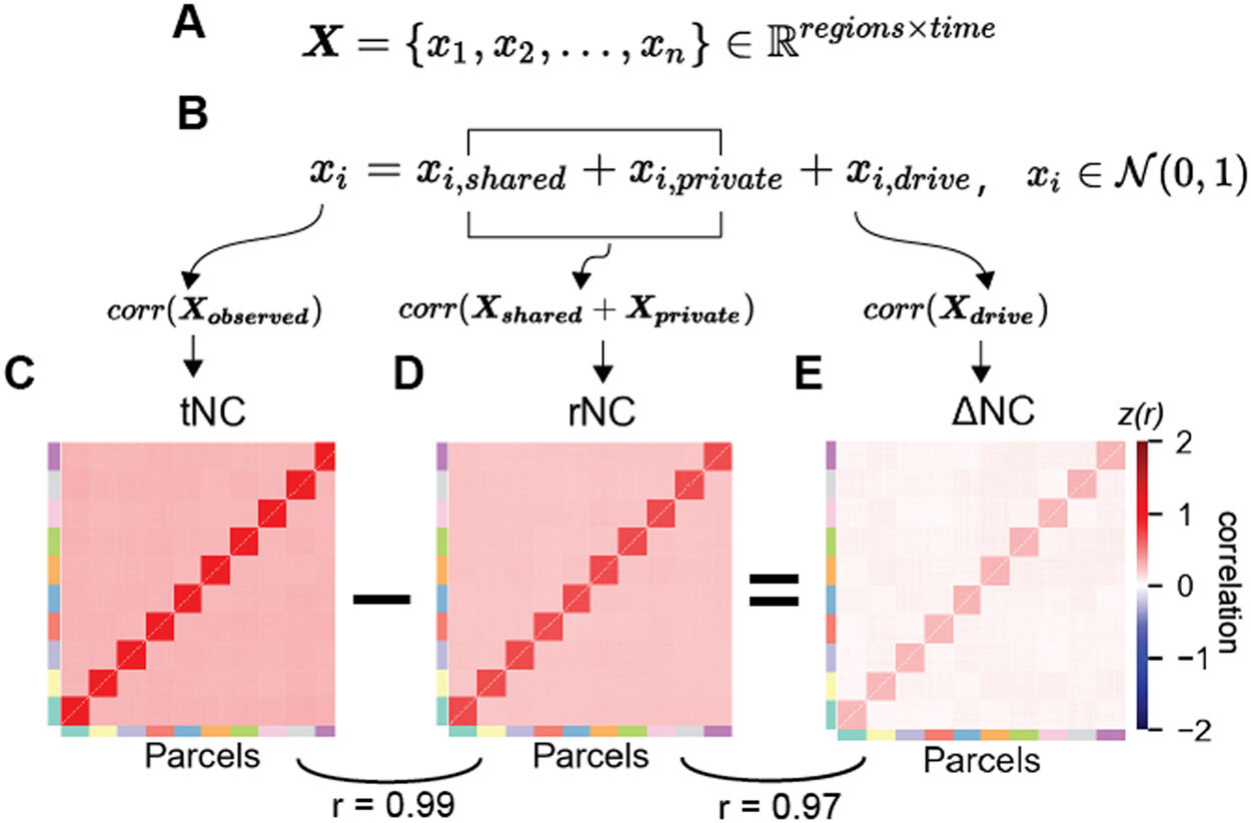
A linear model that predicts neural dynamics during tasks can be decomposed into separable components. (A) We constructed a simple network model with *n* = 360 units and 10 connected networks. (B) To obtain the neural dynamics of a single-brain region *x*_*i*_, linear dynamics were superimposed atop anatomical connectivity with a node’s private dynamics (*x*_*i*,*private*_), a network’s shared dynamics (*x*_*i*,*shared*_), and task dynamics (*x*_*i*,*drive*_). (All *x*_*i*_ were sampled from 𝒩(0, 1).) Under these simple assumptions, the difference between the (C) observed tNC and the (D) baseline rNC yields the (E) stimulus-driven component of correlated activity (i.e., ΔNC) in empirical (or simulated) data.

We constructed a linear statistical model with 360 units and 10 networks (36 units per network). A unit’s activity was determined by the algebraic sum between shared baseline activity (shared between units in the same network), stimulus-driven noise, private noise (for each unit separately), and a globally shared signal (inducing positive correlations between all units; see the Methods section; Figure 3A–B). We found that this model produced positively correlated activity among all pairs of units (mimicking our empirical data), with greater correlation between units within the same network (i.e., shared connections; Figure 3C–D). Critically, when including an additional stimulus-driven component, this primarily increased the magnitude of the correlation primarily between strongly connected units (Figure 3E). This model indicates that under the assumption of linearity, neural units that receive a shared input drive should increase the magnitude of their correlated activity. In other words, with the addition of a new stimulus drive, the state-dependent ΔNC should align with the strength of the underlying SC.

### NC Changes Do Not Typically Align With the Underlying SC in Empirical Data

**Figure 4.**
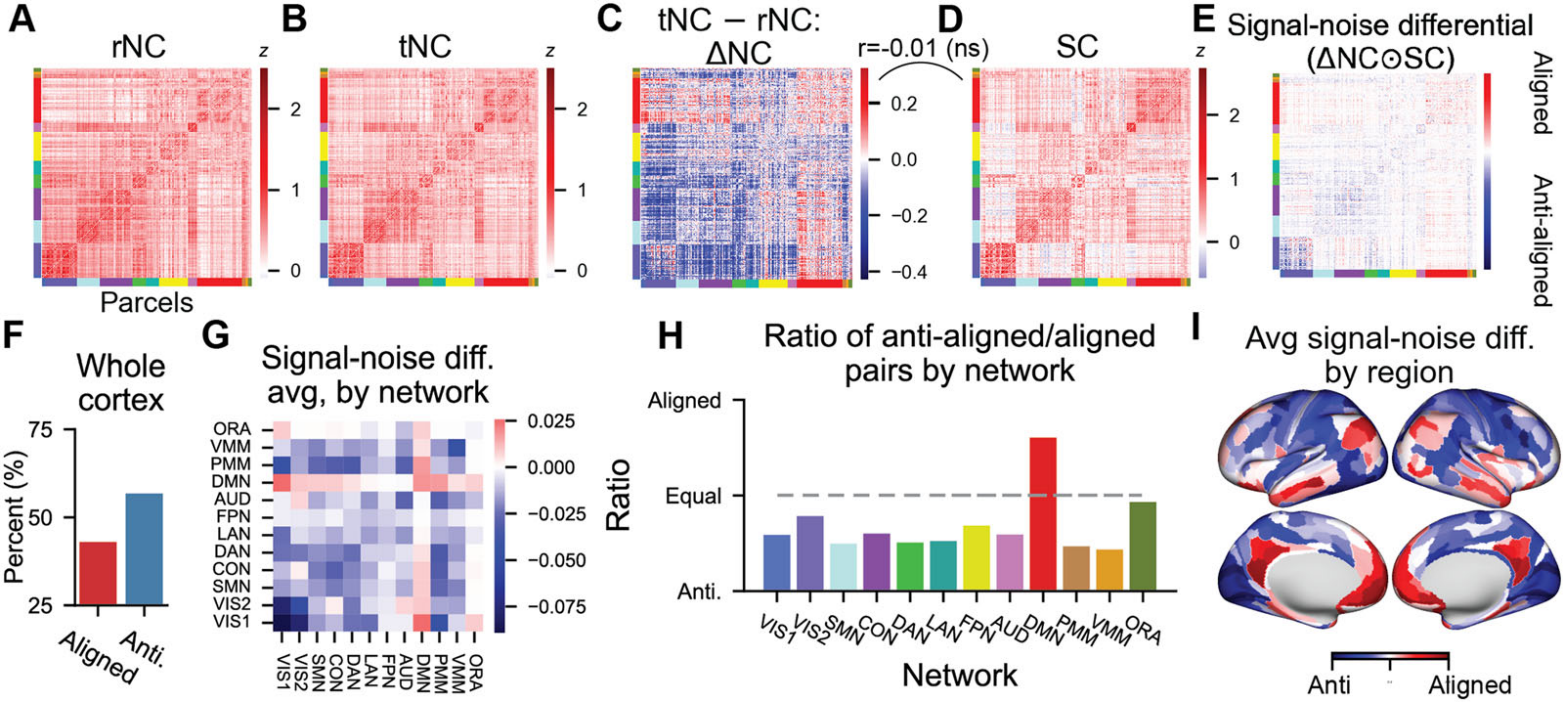
Disambiguating SC and state-dependent ΔNCs in functional brain networks using the signal-noise differential matrix. (A) The rNC and (B) tNC matrix. (C) The tNC vs rNC matrix exhibits widespread correlation reductions. (D) SC matrix, which reflects the task encoding similarity between pairs of regions. (E) The signal-noise differential matrix can be obtained by computing the Hadamard product (element-wise multiplication) of the SC and the ΔNC matrix. The signal-noise differential matrix therefore reflects whether the ΔNC between a pair of regions reflects a change that is aligned (positive) or anti-aligned (negative) with its underlying SC. (F) Percentage of aligned and anti-aligned signal-noise angle pairs across all cortical pairs. (G) The signal-noise differential matrix averaged by network. (H) Percent of aligned versus anti-aligned NCs by each functional network. DMN is the only network that contains more aligned than anti-aligned NC changes. (I) The average of the signal-noise differential matrix for each region (i.e., averaging across columns for each row in panel E).

The statistical model provided an intuition of what should be expected if spontaneous and task-driven variance linearly interact. We next sought to characterize the relationship between the SC and state-related NCs in empirical data. We characterized the rNC, the tNC, and the ΔNC between the two (Figure 4A–C). Consistent with prior work (Ito et al., 2020), we found that the overall change in correlation was dominated by correlation decreases. We computed the signal-noise differential matrix, which we defined as the Hadamard (elementwise) matrix multiplication of the SC matrix (Figure 4D) with the ΔNC matrix (Figure 4C). Note that we calculated the signal-noise differential matrix using the ΔNC matrix since we wanted to understand the impact of state-dependent changes in the NC relative to an ongoing spontaneous activity. Contrary to the statistical model and other studies arguing that NC dynamics are linear (Nozari et al., 2020), we found that most state-related ΔNC did not align with its underlying SC (aligned ΔNC pairs = 42.99%; anti-aligned ΔNC pairs = 56.74%; Figure 4F). This suggests that the majority of state-related ΔNC changes cannot be explained by the linear superposition of a stimulus-driven and spontaneous activity. This finding is also consistent with prior reports indicating that there are a combination of multiplicative and additive fluctuations in ongoing local variability in both rodent electrophysiology (Lin, Okun, Carandini, & Harris, 2015), nonhuman primate electrophysiology (Churchland et al., 2010), and in human fMRI (He, 2013).

We next characterized the network organization of aligned and anti-aligned ΔNCs. While the majority of networks were dominated by anti-aligned ΔNCs, the Default Mode Network (DMN) was instead dominated by aligned ΔNC pairs (Figure 4G–I). The DMN, which primarily consists of the medial prefrontal cortex and posterior cingulate, has previously been shown to suppress its activity during task performance (Dosenbach et al., 2007; Raichle et al., 2001). Prior work characterizing the impact of NCs on neural coding suggest that an aligned signal-noise differential inhibits information coding due to the interference of the correlated noise along the coding (signal) axis (Panzeri et al., 2022). This predicts that the ΔNC increases associated with the DMN (Figure 4C) may inhibit the coding of task-related information. In what follows, we provide a theoretical intuition of why an aligned signal-noise angle inhibits information coding, and we directly test out this theory in fMRI data.

### Interpreting ΔNC Through a Neural Coding Framework

**Figure 5.**
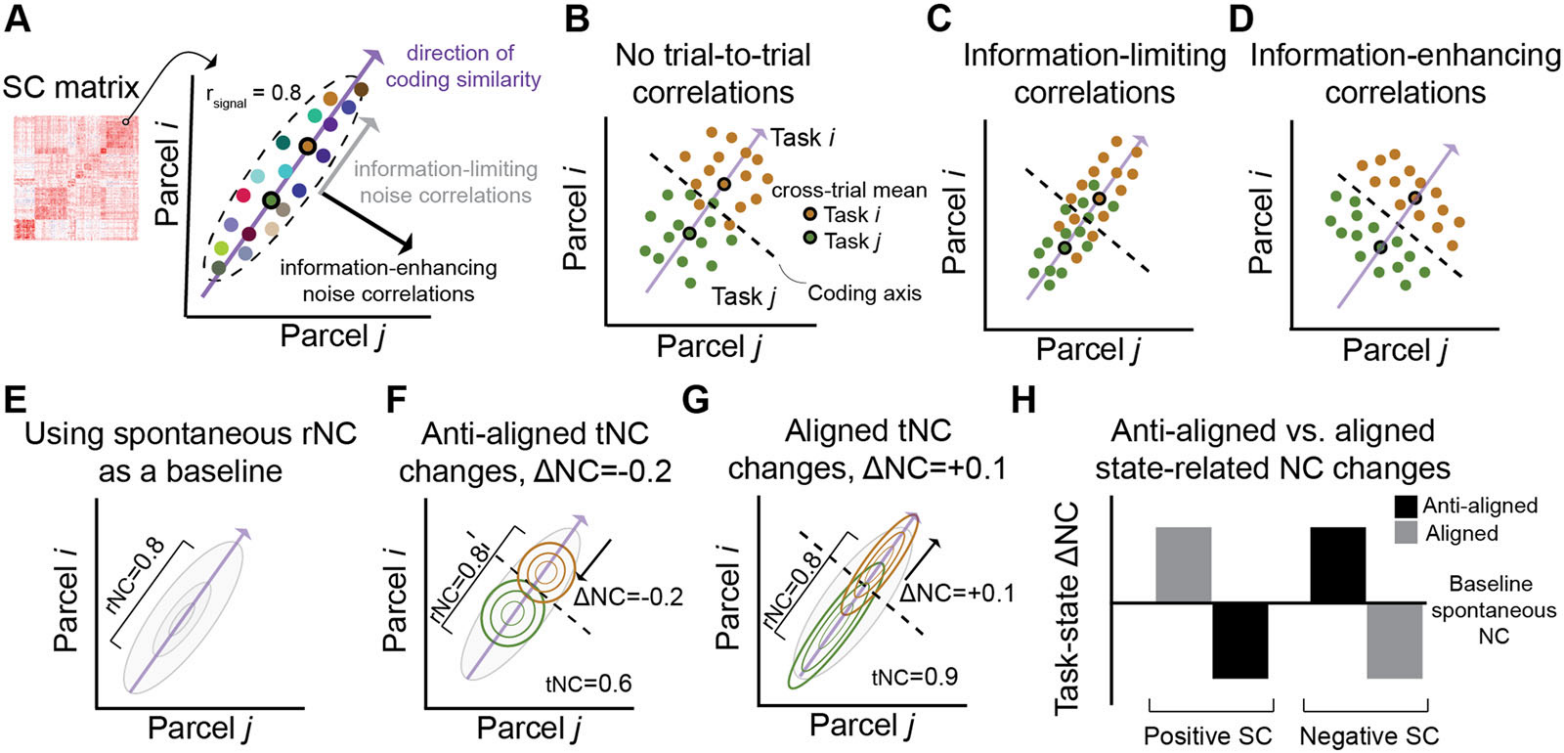
Interpreting ΔNC from an information coding framework. (A) For a pair of brain regions, the SC captures the direction of maximal covariance of the mean activity across many tasks (each colored dot represents the mean activity of a distinct task). tNCs, on the other hand, capture within-task covariability (across events). (B) An example of weak (or no) tNC for two tasks. (C) Prior theories in the neural coding effects of tNC posit that correlations in the same direction as the underlying SC are information limiting. This is because the activity becomes more difficult for a linear decoder to distinguish between the two task conditions. (D) In contrast, tNCs that are in the orthogonal direction as the underlying SC are information enhancing, since the trial-to-trial activity becomes more easily decodable by a linear classifier. (E–G) We modify prior theories to assess how the task-state reconfiguration of tNC impacts information coding relative to the (E) baseline rNC estimate. This modification involves estimating the ΔNC (tNC − rNC). (F) An anti-aligned ΔNC, where the ΔNC is the opposite sign of the SC, thereby reducing noise interference along the SC axis. (G) An aligned ΔNC, where the ΔNC is the same sign of the SC, thereby increasing noise interference along the SC axis. (H) ΔNCs are putatively information limiting or information enhancing based on how the ΔNCs are aligned or anti-aligned with the underlying SC.

There is a rich history in neuroscience of investigating correlated neural activity through the lens of information theory (Abbott & Dayan, 1999; Averbeck, Latham, & Pouget, 2006; Cohen & Kohn, 2011; da Silveira & Berry, 2014; Johnson, 1980; Kohn, Coen-Cagli, Kanitscheider, & Pouget, 2016; Moreno-Bote et al., 2014; Panzeri et al., 2022). Recent theoretical work suggested that the impact of the NC on information coding critically depends on the signs of the SC and NC (da Silveira & Berry, 2014; Moreno-Bote et al., 2014). This intuition can be geometrically described in terms of the signal-noise angle (Panzeri et al., 2022). The signal axis describes the direction of maximal covariance of the mean activity across many tasks/stimuli between a pair of neural units (Figure 5A). The noise axis describes the direction of maximum noise covariance. That is, covariance across repeated instances (e.g., trials or blocks) of the same task/stimuli (Figure 5B–D). Thus, the signal-noise angle describes the angle between these two directions, and reflects whether the NC is information enhancing (orthogonal to the SC) or information limiting (aligned to the SC). However, this initially proposed framework only considers the overall magnitude of the NC, neglecting the impact of spontaneous rNC, which can be used as a baseline. However, prior work in human neuroimaging has shown that the spontaneous correlations estimated during resting-state fMRI are stable and informative (nonzero), reflecting an intrinsic network organization (Gratton et al., 2018). Thus, investigating the impact of NCs on information coding relative to the baseline would shed light on how the brain dynamically reconfigures to support information-enhanced or information-limiting coding between pairs of brain regions.

To assess the reconfiguration of NCs from a baseline state (i.e., rNC) to a task state (i.e., tNC), we made several modifications to prior theories. First, we measured the rNC to establish a baseline (Figure 5E). Next, we measured the tNC and computed the ΔNC (tNC − rNC; Figure 5F–G). If the ΔNC was of the same sign as the underlying SC (i.e., an aligned ΔNC; Figure 5G), this would suggest that the brain dynamically reconfigured such that the tNC would interfere the coding axis. In contrast, if the ΔNC was the opposite sign as the underlying SC (i.e., an anti-aligned ΔNC; Figure 5F), then we would infer that the brain dynamically reconfigures the NC such that the tNC minimizes interference along the SC axis relative to the baseline. Therefore, the product of the SC and ΔNC (Figure 5H)—which we define as the signal-noise differential—serves as a useful estimate to capture how ΔNC impacts information coding.

Though prior work in human neuroimaging has reported more prevalent negative correlations in the rNC matrix, these negative correlations are introduced through a preprocessing technique known as global signal regression (Murphy, Birn, Handwerker, Jones, & Bandettini, 2009). Global signal regression artifactually reduces the mean (across the entire brain) NC to 0, making it difficult to directly compare the impact of magnitude differences across rest and task states. However, here, we derive the rNC and tNC from the same imaging sessions (where rest is interleaved with task), therefore ensuring that differences in NC values cannot be due to different baselines across different imaging runs. This ensures that the comparison of tNC and rNC magnitudes are interpretable. We next test the hypothesis that the relationship between SCs and ΔNCs impacts task information coding in empirical fMRI brain networks.

### The Signal-Noise Differential Determines the Impact of NCs on Task Information Decoding

**Figure 6.**
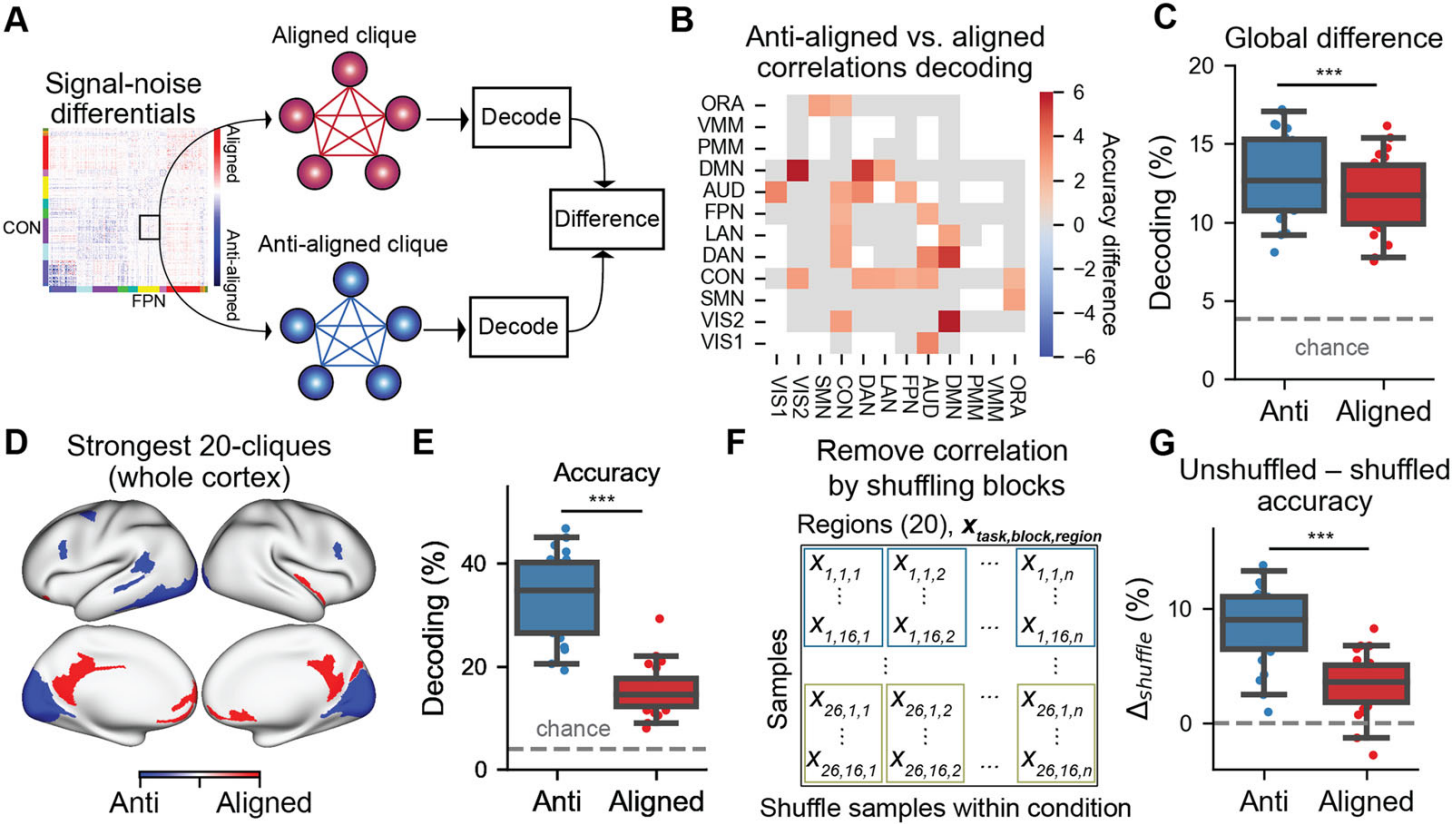
Brain regions with anti-aligned ΔNCs have improved information decoding over aligned ΔNCs. (A) We identified network-matched sets of aligned or anti-aligned ΔNCs by identifying cliques (subnetworks of entirely aligned or anti-aligned ΔNCs). (B) For each pair of networks, we found that sets of brain regions with anti-aligned ΔNCs had significantly higher multitask decoding performance than brain regions with aligned ΔNCs for specific network pairs (FWE-corrected). Note that matrix elements colored in gray had no significant difference. Elements in white were not testable (due to nonexistence of aligned and/or anti-aligned cliques). (C) We computed the average difference for every matrix element in panel (B) for anti-aligned versus aligned cliques, finding that on average, anti-aligned cliques had greater task decodability than aligned cliques. (D) The strongest (highest and lowest) anti-aligned and aligned 20-clique across the entire cortex. (E) The decoding accuracy for the anti-aligned versus aligned ΔNC cliques. (F) We evaluated the impact of NCs by destroying correlated variability when training the linear decoder. This was achieved by randomly shuffling task block structure for each brain region separately. (G) We computed the difference in decoding performance between unshuffled and shuffled conditions. Shuffling task blocks impacted the decoding performance for anti-aligned cliques significantly more than aligned cliques. This is consistent with the hypothesis that the correlation structure of anti-aligned cliques are important for improved task information decoding (since ΔNCs are reconfigured in the opposite direction of SCs). (*** indicates *p* < 0.0001; ** indicates *p* < 0.001; * indicates *p* < 0.05) Box plot bounds define the first and third quartiles (across participants), box whiskers indicate the 95% confidence interval, and the center line indicates the median.

Theoretical work suggests that the signal-noise differential determines how easily task information can be decoded from a set of neural units. To test this empirically, we began by identifying sets of brain regions with entirely aligned or anti-aligned signal-noise differentials (i.e., Figure 4E). We leveraged a technique from network science—clique identification—to identify groups of brain regions with exclusively aligned or anti-aligned signal-noise differentials (Palla, Derényi, Farkas, & Viscek, 2005; Figure 6A). Identifying cliques of either aligned or anti-aligned ΔNCs ensured that all brain regions would either have putatively information-limiting or information-enhancing correlations with each other.

We implemented this by thresholding the signal-noise differential matrix to include either exclusively aligned or anti-aligned NCs and then searching for cliques within these thresholded matrices (see the Methods section). To control for the possibility that identifying cliques would identify brain regions from functionally different networks, we first performed an analysis that identified aligned and anti-aligned ΔNC 5-clique (cliques with five brain regions) for every pair of networks. Identifying both aligned and anti-aligned cliques matched to every network-to-network configuration (e.g., visual to somatomotor network) guaranteed that differences in task decoding were not due to decoding cliques from different functional networks. (We also show corresponding results for 8- and 10-clique; Supporting Information Figure S5).

We directly compared the decoding performance of anti-aligned versus aligned cliques for every pair of networks (Figure 6B). We found that, while not all pair of networks had a statistically significant difference in decoding performance, 24% of network pairs had a significantly higher decoding performance for anti-aligned versus aligned cliques (13/54 network-matched cliques; two-sided Wilcoxon signed-rank test, Bonferroni-corrected *p* < 0.05). (Note that not all network-network pairs contained anti-aligned and aligned 5-clique, and so those networks were excluded; see the matrix elements colored in white; Figure 6B.) Importantly, and as hypothesized, no aligned clique had a greater decoding accuracy than the anti-aligned clique. To obtain a global summary statistic, we computed the average decoding accuracy for all anti-aligned cliques (averaged across all networks) and aligned cliques (Figure 6C). We found that anti-aligned cliques had a significantly higher decoding performance than aligned cliques (accuracy difference = 1.1%, *p* < 10e−06). These findings verify that sets of anti-aligned ΔNCs have improved decodability relative to aligned ΔNCs. Moreover, anti-aligned ΔNCs were overwhelmingly NC reductions (96.4% of all anti-aligned ΔNCs were ΔNC < 0). These results highlight three key insights: (a) The impact of the tNC should be baselined to the spontaneous rNC to infer the impact of NCs on task information decoding; (b) the impact of state-related ΔNCs on task information coding can only be interpreted after knowing the underlying SC; (c) contrary to prior hypotheses in the neuroimaging literature, NC reductions tend to improve task information coding (rather than inhibit communication; for a review, see Gonzalez-Castillo & Bandettini, 2018).

The above task decoding analysis constrained the comparison of anti-aligned and aligned ΔNCs to a specific network pair. However, it is possible that the signal-noise differentials provide useful information about *which* brain regions are involved in optimizing for task information coding. We therefore lifted the constraint of comparing decoding performance between regions within the same networks. Instead, we sought to identify which brain regions are most/least important for task information decoding, by identifying cliques with the strongest anti-aligned/aligned ΔNCs. We identified the 20-clique with the greatest anti-aligned and aligned ΔNCs, as determined by the magnitude of the signal-noise differential. We found that regions with aligned ΔNCs were primarily located in medial prefrontal and posterior cingulate areas (Figure 6D). See also Supporting Information Figures S5I and S5J for a map containing all possible aligned and anti-aligned 20-clique. This was consistent with earlier results, which found that the DMN had a disproportionate number of regions with aligned ΔNCs (Figure 4H). Importantly, when we computed the decoding performance of the aligned 20-clique, it exhibited a significantly lower decoding accuracy than the anti-aligned 20-clique (accuracy difference = 18.24%, *p* < 10e−06; Figure 6E). (We replicated this finding using whole-cortex 15-clique and 25-clique; Supporting Information Figure S5.) This again provides additional evidence that regions with aligned ΔNCs limit task information decoding, while anti-aligned ΔNCs enhance task information coding. Neuroscientifically, these findings also suggest that NCs with the DMN (which are primarily NC increases) inhibit the fidelity of task information coding.

### Destroying Task-State Correlations Impacts the Decodability of Task Information

Supported by theory, our empirical results demonstrate that the alignment of ΔNCs with their underlying SCs impacts task information decoding. However, signal-noise differentials are determined by the relationship of how NCs emerge given the underlying SC. While SC patterns are an intrinsic property of a system (and likely reflect underlying anatomical connectivity; Passingham et al., 2002), the NC is reflected in an ongoing, block-to-block (or trial-to-trial) activity. Thus, we sought to assess if destroying the NCs between brain regions (by shuffling block structure) would impact task decoding.

To destroy the correlated activity between brain regions, we shuffled the block ordering for each brain region separately (Figure 6F; see the Methods section). This removed the effect of tNCs when training a decoder. (Note that in the context of a decoding analysis, shuffling happened on the training set within every cross-validation fold to ensure no leakage between train and test sets; see the Methods section.) We computed the decoder accuracy after removing the NCs for both anti-aligned and aligned cliques. When comparing the difference between unshuffled and shuffled decoder performance (Δ*_shuffle_*), we found that destroying the NC structure of anti-aligned cliques significantly reduced its decoding performance (unshuffled accuracy = 33.6%; shuffled accuracy = 25.3%; *p* < 1e−6; Figure 6G). While shuffling the tNC for aligned cliques also reduced its decoding performance (unshuffled accuracy = 15.4%; shuffled accuracy = 11.9%; *p* < 1e−5; Figure 6G), removing the effect of NCs had a significantly greater impact on the anti-aligned ΔNCs (Δ*_shuffle_* anti-aligned = 8.4%; Δ*_shuffled_* aligned = 3.4%; *p* < 1e−5; Figure 6G). These empirical findings are consistent with the hypothesis that the tNC of anti-aligned cliques significantly enhances information coding relative to aligned cliques, and demonstrates the information-coding relevance of tNC changes.

## DISCUSSION

We leveraged insights from neural coding to interpret large-scale, task-state correlation changes in human fMRI data. We first characterized the SCs and NCs of human fMRI data using a multitask dataset with 26 cognitive tasks, finding that SCs had greater network modularity and segregation than the commonly used rNC matrix. This suggested that the SC may have greater utility than the rNC in identifying functional specialization across cortical regions. Next, we sought to understand how NCs emerge from underlying network dynamics. We constructed a linear statistical model to gain an intuition of how state-dependent NCs interact with each other. This model revealed that—under the assumption of linear dynamics—the tNC should emerge as the algebraic sum of spontaneous background activity and stimulus-specific activity. This implied that stimulus-specific NCs should always align with the underlying SC. In contrast to this model, we did not find this pattern in empirical NCs. Instead, a majority of ΔNCs were anti-aligned with the underlying SCs. This led us to interpret these anti-aligned ΔNCs through a neural coding perspective, which predicts that anti-aligned ΔNCs should improve task information coding. This is because the NCs are reconfigured to avoid interference along the SC axis. Indeed, when testing this prediction in empirical data, we found that anti-aligned ΔNCs had significantly higher task decoding accuracies than ΔNCs that were aligned with their underlying SCs. Together, these findings provide a task information coding perspective to interpret task-state correlation changes in human functional brain networks.

In the human neuroimaging literature, studies of interregion communication are viewed through the lens of “FC.” While FC is a broad umbrella term that incorporates a variety of techniques (Cliff, Bryant, Lizier, Tsuchiya, & Fulcher, 2023; Frässle et al., 2018; Friston, 2011; Reid et al., 2019; Sanchez-Romero & Cole, 2021), the most commonly used measure is the Pearson correlation—the same metric used in computing spike count NCs. Yet despite the use of identical statistical metrics across the human neuroimaging and neurophysiology, the frameworks for interpreting correlations diverge. On one hand, human neuroimaging studies often analogize the strength of correlation with the strength of “communication” (for a review of the literature, see Gonzalez-Castillo & Bandettini, 2018). On the other hand, NCs are typically viewed through the lens of how they impact task information coding (Abbott & Dayan, 1999; Cohen & Kohn, 2011; Panzeri et al., 2022). Empirically, we found that the majority of NCs that enhance information coding—anti-aligned ΔNCs—tend to be tNC decreases (96.4% of anti-aligned ΔNCs are decreases). This finding places these two views at odds, since prior interpretations of tNC reductions have been interpreted as “reduced” or segregated communication among brain regions (Rubinov & Sporns, 2010; Wig, 2017). Here, we suggest that the neural coding perspective provides a parsimonious explanation for why reduced tNCs are widespread and enhance task coding: The anti-alignment of the ΔNC with the SC minimizes the amount of signal interference between the two brain regions. Given that the majority of brain regions have a positive SC (Figure 2B), it follows that the majority of ΔNCs should be reductions to enhance task information coding among brain regions.

There are several conceptual and methodological differences in neural correlation studies between fMRI and electrophysiology literatures relevant to our study. First is the use of the term noise. In neural spiking data (particularly after spike sorting), the term neural noise implies neural activity that cannot be accounted for by experimenter-controlled parameters (e.g., spontaneous activity). In fMRI, noise can emerge from a multitude of sources in addition to spontaneous activity, such as physiological noise, thermal noise, and participant motion. While we employed noise removal methods to triangulate the neural activity of interest (i.e., spontaneous neural activity; Ciric et al., 2017), it is possible that the effect of the NCs we measured were not necessarily purely neural in nature. Nevertheless, it will be important for future work—such as approaches that use simultaneous fMRI with neurophysiology (Ma et al., 2016; Shahsavarani et al., 2023) and those that leverage better individualized estimation methods for fMRI activity (Prince et al., 2022)—to directly verify whether the impact of NCs on fMRI decoding originates from neural sources. Second, we used a resting-state activity to model baseline neural correlations, whereas electrophysiology studies typically use a baseline control condition. This makes it difficult to fully determine whether changes in neural correlations are due to the effects of an unconstrained neural state. It will be pertinent for future work to assess the impact of NC structure during unconstrained and constrained states. Finally, most studies in the fMRI literature typically estimate tNC using adjacent time points during task performance blocks (Cole et al., 2014; Krienen et al., 2014), rather than the across-trial correlations commonly employed in electrophysiology and also in this study. However, computing the correlation across adjacent timepoints within a task block can make it difficult to disambiguate signal and noise sources, if proper removal of the mean task effect is not performed (Cole et al., 2019). The present approach disambiguates SC and NC measurements by isolating the cross-block mean and cross-block variance by obtaining block-to-block activation estimates separately. Importantly, this is the common approach to calculating SCs and NCs in the neurophysiology literature (Cohen & Kohn, 2011). Nevertheless, to demonstrate the generality of the statistical inferences made here, we found a high correspondence between the ΔNC matrices when computing tNC across timepoints during task blocks (Supporting Information Figure S1). (This is the commonly used approach to estimating tNC in fMRI neuroimaging.) Together, these findings suggest that differences in tNC calculation should not influence the present conclusions.

Our findings are also widely consistent with prior studies across subfields in neuroscience that find widespread decorrelations during task states. These studies revealed that during task and attentional states, correlations are reduced among pairs of neurons (Cohen & Maunsell, 2009), cortical regions accessed with wide-field calcium imaging data (Pinto et al., 2019), mean-field multi-unit recording across cortical regions in nonhuman primates (Ito et al., 2020), and human fMRI correlations (Ito et al., 2020). While prior literature has demonstrated that spike count correlations impact information coding in nonhuman primates (Cohen & Maunsell, 2009; Ni, Huang, Doiron, & Cohen, 2022), it was an open question as to whether these intuitions would scale to larger spatial organizations and broader cognitive tuning curves. Our findings affirm that the generic statistical principles developed to understand neural coding in spiking units are translatable to different data modalities, and naturally scale up to broader spatial levels of organization. However, the current study only takes into account task-general changes to NCs, rather than task-specific NC changes. While prior work in nonhuman primate spike recordings suggest that NCs change to support task coding *in general* (rather than optimally for each task; Ni et al., 2022), it will be important for future studies to investigate the contribution of task-general NC changes versus task-specific NCs to support task information coding.

The present findings, as well as current limitations, open new questions that future studies can explore. First, while the finding that anti-aligned correlations improve the task decodability of those brain regions and networks, it is unclear how this optimized information is implemented and used by the brain. Which downstream brain regions read out this information? What are the biophysical mechanisms that produce anti-aligned ΔNCs? Future work can build on this work to investigate how optimized task information is used and implemented by the brain (De-Wit, Alexander, Ekroll, & Wagemans, 2016). Second, the intuitions behind how the signal-noise differential impacts task coding were developed for two dimensions (i.e., two regions or neurons; Figure 5; da Silveira & Berry, 2014; Moreno-Bote et al., 2014). While we demonstrate that these intuitions generally apply for more than just two regions (e.g., improved decoding for anti-aligned cliques), it is not explicitly clear how these intuitions generalize to greater dimensions. It will be important for future work to develop theory and measures (beyond just the signal-noise differential/angle) beyond two dimensions.

Third, there is a rich fMRI literature on time-varying FC and dynamic coactivation patterns at rest (Hutchison et al., 2013; Lurie et al., 2020). However, in our analysis, we assumed the rNC to be stable, as other studies have shown the static and dynamic FC represents a largely similar information (Matkovič, Anticevic, Murray, & Repovš, 2023). Nevertheless, it will be important for future studies to assess the impact of large-scale spontaneous dynamics and how they might influence neural coding. Finally, interpreting the impact of NCs on task coding requires knowledge of the underlying SC. In many cases and existing datasets, however, identifying the SC is infeasible, since it requires many tasks and conditions. It will be interesting for future work to develop techniques to approximate the SC without acquisition of task data, such as anatomical connectivity fingerprinting, which has been thought to define the functional tuning of local brain regions (Passingham et al., 2002).

In conclusion, we disambiguate the SC and NC in large-scale human functional brain networks using a multitask fMRI dataset, and characterize the impact of NCs on task information coding. This work bridges the disparate fields of the spike count correlation analyses (typically carried out in nonhuman animals) with the emerging field of task-state functional connectomics in humans. Importantly, our findings place functional connectomics within a broader framework of neural coding, demonstrating the impact of the task-state FC for task coding. We hope these findings spur future investigations into understanding the properties of task information coding in large-scale human brain networks.

## METHODS

### Multidomain Task Battery Dataset

Portions of this section are paraphrased from the dataset’s original publication’s Methods section (King et al., 2019) and a prior study we used to investigate multitask cortical representations (Ito & Murray, 2023).

We used the publicly available multidomain task battery (MDTB) dataset, which was originally published to study the functional (task) boundaries of the human cerebellum (King et al., 2019). The dataset contains both resting-state and task-state fMRI data for 24 subjects collected at Western University (16 women, 8 men; mean age = 23.8 years, *SD* = 2.6; all right-handed; see King et al. [2019] for exclusion criteria). All participants gave informed consent under an experimental protocol approved by the institutional review board at Western University.

The MDTB dataset collected data during 26 cognitive tasks and up to 45 different task conditions for each participant. Tasks were grouped together in two sets (set A and B; Figure 1E). Each participant first performed all tasks in set A and returned for a second session to perform tasks in set B. Each task set consisted of two imaging runs. Half of the subjects had sessions separated by 2–3 weeks, while the other half had sessions separated by roughly a year. A separate resting-state scan with two 10-min runs each was collected for 18/24 subjects. (This resting-state scan was independent of the “rest” block in the task imaging sessions.)

The MDTB dataset was designed to target diverse cognitive processes. Sets A and B contained eight overlapping tasks (e.g., theory of mind and motor sequence tasks) and nine tasks unique to each set (Figure 1A). Both sets contained 17 tasks each. Further details about the experimental tasks and conditions have been previously reported in the original dataset publication (see Supplementary Table 1 of King et al., 2019; https://static-content.springer.com/esm/art%253A10.1038%252Fs41593-019-0436-x/MediaObjects/41593_2019_436_MOESM1_ESM.pdf).

Tasks were performed once per imaging session. Tasks were presented in an interleaved block design. Task blocks began with a 5 s instruction screen, followed by 30 s of continuous task performance. Eleven out of 26 tasks were passive and required no motor response (e.g., movie watching). Tasks that required motor responses were made with either left, right, or both hands using a four-button box using either index or middle fingers. All tasks (within each set) were performed within a single imaging run, ensuring a common baseline between tasks for all participants.

### fMRI Preprocessing

Portions of this section are paraphrased from a prior study using a similar preprocessing strategy (Ito & Murray, 2023).

fMRI data were minimally preprocessed using the Human Connectome Project (HCP) preprocessing pipeline. The HCP pipelines were implemented within the Quantitative Neuroimaging Environment & Toolbox (QuNex, version 0.61.17; Ji et al., 2022). The HCP preprocessing pipeline consisted of anatomical reconstruction and segmentation, EPI reconstruction and segmentation, spatial normalization to the MNI152 template, and motion correction.

### fMRI Task Activation Estimation

Portions of this section are paraphrased from a prior study using a similar preprocessing strategy (Ito & Murray, 2023).

We performed a single-subject beta series regression (Rissman et al., 2004) on fMRI task data to estimate parcelwise activations using the Glasser atlas (Glasser et al., 2016). Each task block (30 s) was modeled with a separate task regressor. The instruction period prior to the task block was not included. Thus, the number of task regressors was equivalent to the total number of task blocks per imaging session. Each task regressor was modeled as a boxcar function from the block onset to offset (0 indicate off, 1 indicate on) and then convolved with the SPM canonical hemodynamic response function to account for hemodynamic lags (Friston et al., 1994). We used the coefficients of each regressor as the activation for each task block. Task General Linear Models (GLMs) were implemented in Python using the LinearRegression function within scikit-learn (version 0.23.2) in Python (version 3.8.5). Task GLMs were performed simultaneously with nuisance regression. This was done in an effort to isolate nonneural noise sources when estimating task activations and spontaneous activity. Importantly, the task was modeled simultaneously with noise parameters, as prior studies have shown that removing noise parameters in sequence can artificially induce artifacts (Lindquist, Geuter, Wager, & Caffo, 2019). The noise parameters we removed were six motion parameters, their derivatives, and the quadratics of those parameters (24 motion regressors in total). We removed the mean physiological time series extracted from the white matter and ventricle voxels, their derivatives, and the quadratics of those time series (eight physiological nuisance signals). In total, there were 32 nuisance regressors. For task fMRI data, nuisance regressors were included simultaneously with task regressors to extract the task activation estimates described below.

### SC and NC Estimation

The SC between two brain regions was computed through the following steps. The mean activation of each task was computed by averaging the blockwise GLM coefficients for that task. This resulted in 26 task activations for every brain region. The SC was then computed as the across-task correlation. Note that since 8 of the 26 tasks were performed in both task sets (i.e., set A and set B), we only included data from one of the task sets. This helped to control the data imbalance across different tasks, ensuring that every task had an equal number of blocks when calculating the mean task activation (16 blocks).

The NC for two brain regions was estimated for each task separately. The NC estimation that we performed is identical to task-state FC calculation using a beta series regression (Rissman et al., 2004).

To estimate the tNC using task blocks, block-to-block activation coefficients were obtained for each task separately. Each task had 16 blocks across all imaging sessions. The NC for a pair of regions was the across-block correlation *within* a task. Since there were 26 tasks, there were 26 NCs for every pair of brain regions. We averaged the NC across all tasks (excluding the rNC) to obtain a task-general tNC matrix. The rNC was computed using the resting-state blocks during the task imaging session. To verify that the beta series regression approach to calculating the NC is similar to other NC calculation approaches (i.e., using timepoints within a task block), we also compared NC estimates using correlations across timepoints (Supporting Information Figure S1). Importantly, rNCs, tNCs, and ΔNCs were highly similar to each other despite differences in how they were estimated (block-to-block activity vs. timepoint-to-timepoint activity). This indicated that task coding properties of NCs evaluated here generalize to both block-to-block and timepoint-to-timepoint NC estimates.

Note that the timepoint-to-timepoint NC estimation performed in Supporting Information Figure S1 is consistent with prior approaches to calculating the NC (Cole et al., 2019; Ito et al., 2020). Specifically, for each task, we fit a finite impulse response model (across blocks of the same task) to remove the mean-evoked response (which includes the hemodynamic response). This approach flexibly removes the mean-evoked response, while taking into account each brain region’s idiosyncratic hemodynamic response shape. We removed hemodynamic effects of up to 20 s after a task block offset. This ensured that the task signal from the previous block would not overlap (interfere) with task signals in the next block. NCs were then calculated on the residual time series. This approach ensured that NCs were not conflated by the mean (i.e., signal) response. We also did not directly compare the tNC with rNC using data from the separate resting-state fMRI scan. This was because the data obtained during the task sessions spanned over 5 hr, whereas the separate resting-state scan was only 10 min long. This made it impossible to perform a direct comparison of task- to rest-state fMRI using the same temporal intervals in this dataset.

### Network Analysis

We performed both network-style (Rubinov & Sporns, 2011) and gradient-style (Huntenburg et al., 2018) analyses on SC and NC matrices. Network-style analysis included computing the network modularity and network segregation of SC and rNC matrices with respect to a previously published functional network partition (Ji et al., 2019).

We used an undirected signed modularity metric that calculates modularity with respect to a provided network partition (Rubinov & Sporns, 2011). We use the asymmetric variant that treats positive and negative values differently (i.e., positive values link nodes within a module, and negative values dissociate nodes between modules). Modularity was calculated as follows:

$$Q^{*}=Q^{+}+\frac{v^{-}}{v^{+}+v^{-}}Q^{-},$$

where *Q *^±^ is defined as follows:

$$Q^{\pm }=\frac{1}{v^{\pm }}\sum_{ij}\left(w_{ij}^{\pm }-e_{ij}^{\pm }\right)\delta_{M_{i}M_{j}},$$

$w_{ij}^{\pm }$ is the connection weight (positive or negative values only), *v*^±^ = ∑ $w_{ij}^{\pm }$, $e_{ij}^{\pm }$ is the chance-expected within module connections defined as $e_{ij}^{\pm }=\frac{s_{i}^{\pm }s_{j}^{\pm }}{v^{\pm }}$, where $s_{i}^{\pm }=\sum w_{ij}^{\pm }$, *δ*_*M*_*i*_*M*_*j*__ = 1 when *i* and *j* are in the same network module and *δ*_*M*_*i*_*M*_*j*__ = 0 otherwise. The code was implemented using the brain connectivity toolbox (bctpy version 0.5.0).

Network segregation was measured as the difference between within-module and between-module weights, divided by within-module weights (Chan, Park, Savalia, Petersen, & Wig, 2014). Segregation was first calculated for each region separately and then averaged across all regions. Segregation of a region *i* was computed as follows:

$$s_{i}=\frac{x_{\text{in}}-x_{\text{out}}}{x_{\text{in}}},$$

where *x*_*in*_ is the within-module weights for region *i* and *x*_*out*_ is the between-module weights.

Gradient-style analysis was computed by performing a principal components analysis (PCA) on either the SC or NC matrices. SC and NC matrices were thresholded to retain only 20% of the strongest correlations prior to calculating gradients. The PCA was implemented using scikit learn’s PCA function (sklearn.decomposition.PCA, version 1.0.2).

### Linear Statistical Network Model

We used a statistical model to predict how task-related variability influences a baseline spontaneous activity. We partitioned 300 nodes into 10 networks (30 nodes each). Networks were fully connected with a weight of 1. Since the rNC typically exhibits positive correlations among all pairs of regions, we introduced a globally shared signal. Specifically, a region’s activity *x*_*i*_ was determined as the linear sum of Gaussian distributions (10,000 samples), *X* ∼ *N*(0, 1):

$$x_{i}=x_{\text{shared}}+x_{\text{private}}+{s_{\text{drive}}}^{*}0.75+g^{*}0.75,$$

where *x*_*shared*_ is a shared time series among nodes within a network, *x*_*private*_ is a unique time series for *x*_*i*_, *s*_*drive*_ is the stimulus-related variance (set to 0 in the baseline spontaneous case), and *g* is the global variance shared by all nodes.

### Task Decoding Analyses

#### Clique identification.

We identified cliques of aligned and anti-aligned signal-noise differentials. This was to test the impact of signal-noise differentials on task information decoding. Cliques are a subnetwork of a graph that are fully connected (Sizemore et al., 2018). This means that every node is connected to every other node in that subnetwork. Using the signal-noise differential matrix (Figure 6A), we identified aligned and anti-aligned cliques by creating thresholded matrices of exclusively aligned and anti-aligned region pairs, respectively. This ensured that when performing decoding analyses on a set of regions, every pair of region was either aligned or anti-aligned. This was important given that, from a neural coding perspective, aligned signal-noise differentials would be information limiting relative to baseline correlations, while anti-aligned signal-noise differentials would be information enhancing (Figure 5).

We identified aligned and anti-aligned 5-clique for every pair of functional network configuration (e.g., region sets between the DMN with the frontoparietal network; Visual 1 network to the somatomotor network). Matching aligned and anti-aligned 5-clique to the specific network configuration controlled for the possibility of inherent differences in decoding performance when identifying cliques from different networks (e.g., there might be intrinsic differences when comparing the decoding performance of an aligned clique in the DMN vs. the Visual 1 network). However, aligned and anti-aligned 5-clique did not exist for all network pairs. These network pairs were therefore excluded from the analysis, since aligned and anti-aligned decoding performances could not be directly compared. In supplementary analyses, we also show that our findings generalize to 8-clique and 10-clique (Supporting Information Figure S5).

In addition, we identified 20-clique with the strongest aligned and anti-aligned 20-clique (Figure 6D). Strongest was defined as having the greatest negative or positive average values within an aligned or anti-aligned 20-clique (using the signal-noise differential matrix; Figure 6A). Note that while the aligned and anti-aligned 20-clique were in spatially disjoint regions, decoding performance was appropriately controlled for by removing the effect of correlated variability (e.g., Leavitt, Pieper, Sachs, & Martinez,-Trujillo, 2017; Figure 6F–G), as discussed in the next subsection.

Clique identification was carried out using the python package NetworkX (networkx.find_clique function; version 2.5). To make identifying cliques more tractable, the signal-noise differential matrix was thresholded to retain only the top 20% positive or negative values. Note that we also replicated these findings using 15- and 25-clique (Supporting Information Figure S5). For the 25-clique analysis, we thresholded the signal-noise differential matrix to the top 40% of positive or negative values.

#### Decoding analyses.

To assess the role of NCs on task information coding, we performed a multitask (26-way) linear decoding analyses. Decoding analyses were performed within subjects, using the blockwise activations of every task. There were 26 tasks with 16 blocks per task (416 samples per subject). We performed a leave-one-out cross-validation, cross-validating across blocks. Samples in the training set were bootstrapped (20 samples per task type, with replacement). Prior to fitting the linear decoder on the training sets, samples in the training set were feature-normalized (*z*-normalized), and samples in the test set were also feature-normalized using the mean and standard deviation estimated from the training set (to avoid train-test leakage). A linear decoder was fit using logistic regression and was implemented using scikit-learn (version 1.0.2).

To evaluate the effect of correlated variability on aligned and anti-aligned cliques, we performed a follow-up analysis that removed the impact of NCs on linear decoding. This was implemented by shuffling the ordering of task blocks for each brain region and each task type separately (see Figure 6F). This was done on the training set within each cross-validation fold. Shuffling blocks for each brain region separately removed the contribution of NCs on training a linear decoder.

Note that while prior studies suggest that the best practices for decoding analyses employ a five- or 10-fold cross-validation (Varoquaux, 2018), we used a leave-one-out cross-validation approach to maximally assess the impact of a correlated activity (within the training set) on decoding performance. (Note exactly one sample from each task was left out from the training set, such that the test set had 26 samples in total.) Moreover, we were not focused on making inferences on decoding performance relative to chance. Instead, we were interested in assessing how a correlated activity (within the training set) impacted decoding performance for aligned and anti-aligned cliques, and how a shuffling correlated activity would (within the training set) impact overall decoding performance. If NCs had no impact on decoding performance, shuffling the block structure in the training set would have no impact on task decoding.

### Data Visualization

All graphical plots were visualized using seaborn (version 0.11.2; Waskom, 2021). All cortical surface plots were visualized using surfplot (version 0.1.0; Gale et al., 2021; Vos de Wael et al., 2020).

## ACKNOWLEDGMENTS

This project was supported by NIH grant R01MH112746 (J.D.M.), NSF NeuroNex grant 2015276 (J.D.M.), and a Swartz Foundation Fellowship (T.I.). The authors acknowledge the Yale Center for Research Computing at Yale University for providing access to the Grace cluster and associated research computing resources.

## SUPPORTING INFORMATION

Supporting information for this article is available at https://doi.org/10.1162/netn_a_00402.

## AUTHOR CONTRIBUTIONS

Takuya Ito: Conceptualization; Formal analysis; Investigation; Methodology; Software; Visualization; Writing – original draft; Writing – review & editing. John D. Murray: Conceptualization; Funding acquisition; Project administration; Resources; Supervision; Writing – review & editing.

## FUNDING INFORMATION

John D. Murray, National Institute of Mental Health (https://dx.doi.org/10.13039/100000025), Award ID: R01MH112746. John D. Murray, National Institute of Mental Health (https://dx.doi.org/10.13039/100000025), Award ID: P50MH109429.

## CODE AND DATA AVAILABILITY

All data in this study have been made publicly available on OpenNeuro by King and colleagues (2019): https://openneuro.org/datasets/ds002105.

All codes related to this study are publicly available (https://github.com/ito-takuya/signalNoiseFC). Analyses and models were implemented using Python (version 3.8.5).

## Supplementary Material



## TECHNICAL TERMS

Functional connectivity: The statistical relationship (often measured with correlation) between the functional activity between brain regions.
Signal correlation: The correlation (similarity) of the tuning profiles or cognitive functions between brain regions.
Noise correlation: The temporal correlation between neural units across repeated events of the same task or stimulus.
Signal-noise differential: The product of the noise correlation change with the underlying signal correlation between two units.
Anti-aligned correlation: Noise correlations that change in the opposite direction as their signal correlation (negative signal-noise differential), facilitating better task decoding.
K-clique: A set of fully connected units (e.g., brain regions).
