# Supplementary material for: The impact of functional correlations on task information coding
Source: Netw Neurosci. 2024 Dec 10;8(4):1331–54. doi: 10.1162/netn_a_00402 (PMC11675092; doi:10.1162/netn_a_00402)
Supplement: Supplementary file 1 [file netn-8-4-1331-s001.pdf]

Supplementary Figures

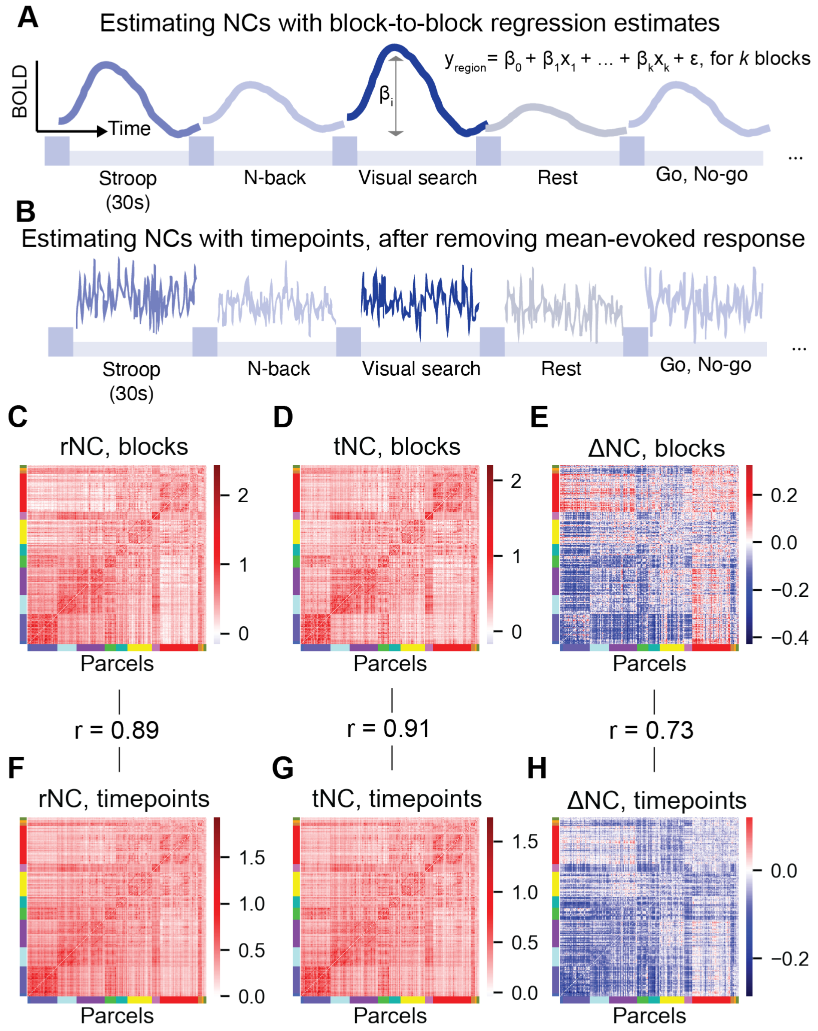

Supplementary Figure 1. NCs computed using block-to-block activity estimates versus timepoint-to-timepoint estimates reveal quantitatively similar NC estimates. a) To estimate NCs using block-to-block estimates, we performed a beta series regression. In a beta series regression, every block (or trial) has its own independent regressor. Every block/trial therefore has its own activity estimate. (Image is a schematic.) b) To compare NCs using the more traditional approach, we estimated NCs using correlations estimated across timepoints within task blocks. To ensure task-driven variance/noise was not conflated with the mean-evoked (i.e., signal) response, we performed a finite impulse response model across all blocks for each task type separately (Cole et al., 2019). This ensured that NCs were computed using the background task-driven variance. c) The rNC matrix computed using rest blocks (as implemented in the main text). d) The tNC matrix computed using task blocks, averaged across all tasks (as implemented in the main text). e) The ΔNC matrix using block-wise NC estimates (as implemented in the main text). f) The rNC matrix computed as the correlation across timepoints. Resting-state blocks were first concatenated across all imaging sessions for a participant. The rNC was then computed on the concatenated time series. g) The tNC matrix computed as the correlation across timepoints. Blocks for each unique task were first concatenated for each participant. tNC was computed for each task, and then averaged across all tasks to obtain a task-general NC matrix. h) The ΔNC matrix using timepoint-to-timepoint NC estimates. Despite being computed using different approaches (with varying amounts of data per NC), rNCs (r=0.89), tNCs (r=0.91), and ΔNCs (r=0.73) were highly similar across these approaches.

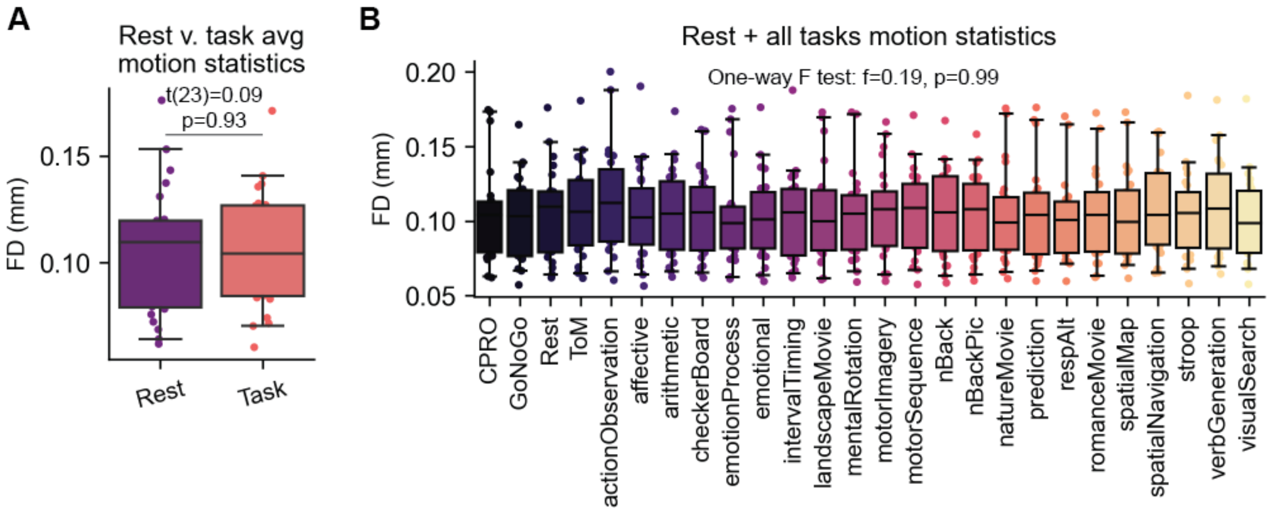

Supplementary Figure 2. Motion statistics for each subject across rest and task states. a) We computed the framewise displacement (FD; Power et al. (2012)) for all timepoints during rest and task blocks. We found no significant difference between the average FD between rest and task states. b) The average FD during the blocks across rest and each task individually. We performed a one-way F test to assess if the FD of a given state was statistically different from the FD of all other states. We found no statistically significant deviation of FD across all states. Box plot bounds define the first and third quartiles of the (across participant) distribution, box whiskers indicate the 95% confidence interval, and the center line indicates the median.

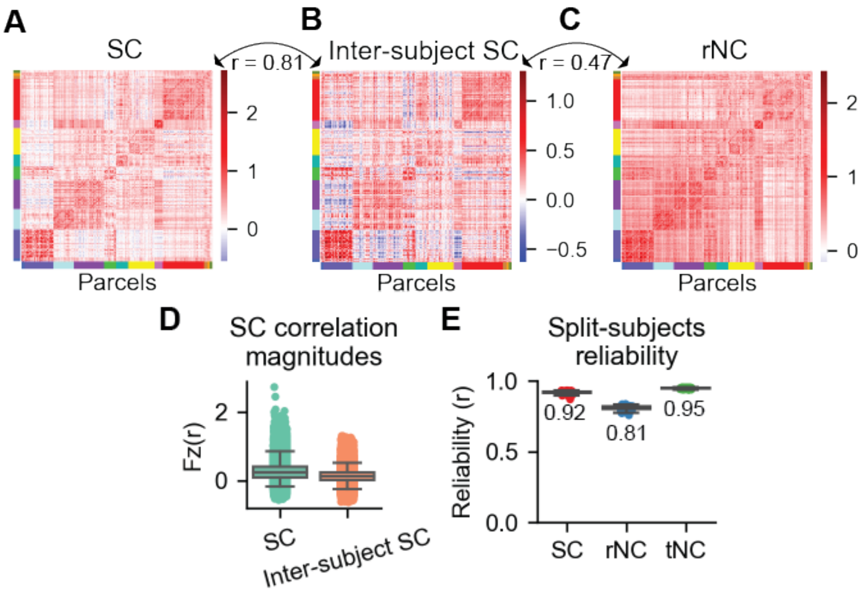

Supplementary Figure 3. Reliability of the SC, rNC, and tNC matrices. a) The group-averaged SC matrix (same as in the main text). b) The inter-subject SC matrix. The inter-subject SC removes the potential confound of noise sources that are idiosyncratic to the participant and/or scan session (Kim et al., 2018). This was calculated by taking one subject's multi-task activation vector for a single brain region, and then correlating that vector with the group-averaged (excluding that one subject) multi-task activation vector from all other brain regions. This approach exclusively captures SC at the group-level. We found that the group-averaged SC matrix and the inter-subject SC matrix maintained a high correspondence (r=0.81), suggesting that in general, the organization of the group-averaged SC captures the organization of signal correlations that are not subject-specific. c) The group-averaged rNC matrix (same as in main text), for comparison. d) The distribution of correlation values between the group-averaged SC (a) is wider than the distribution of the inter-subject SC (b). This is because inter-subject SC removes sources of correlated variability that are idiosyncratic to an individual. Boxplots reflect the distribution across all pairs of correlations. Box plot bounds define the first and third quartiles, box whiskers indicate the 95% confidence interval, and the center line indicates the median. e) Group-averaged SC, rNC, and tNC measurements are overall reliable. We performed a splits subject analysis, randomly sampling half the subjects SC/rNC/tNC, and correlating it with the SC/rNC/tNC of the other half, respectively. Across participant splits, SC, rNC, and tNC reliability was above r=0.81. rNC had the lowest reliability, which is likely due to the fact that rNC correlations were computed using only 16 samples per pair of parcels. (tNC is computed by correlating 16 samples per pair of parcels per task, and then averaged across tasks. SC is computed by estimating the average activation for each task, and then correlating across 25 tasks.) We bootstrapped 100 random splits; box plot bounds define the first and third quartiles of those 100 splits, box whiskers indicate the 95% confidence interval, and the center line indicates the median.

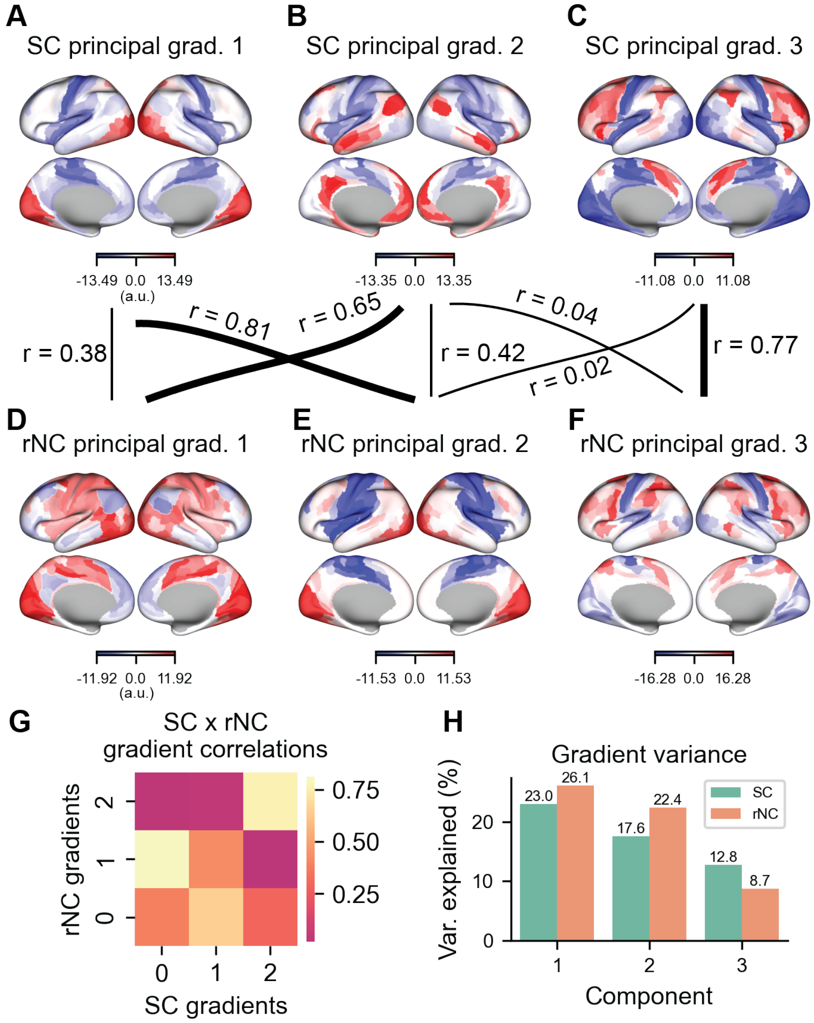

Supplementary Figure 4. Detailed comparison of the first three SC and rNC gradients. a) The first, b) second, and c) third SC gradient. d) The first rNC gradient, which has highest similarity to the second SC gradient. e) The second rNC gradient, which has highest similarity to the first SC gradient. f) The third rNC gradient, which has greatest similarity to the third SC gradient. Together, these findings suggest that the first three dimensions of SC and rNC are similar, but that the first two components are flipped in SC and rNC. Note that correlation values reflect the absolute value, since the orientation of PCA loadings are arbitrary. g) All pairwise correlations (absolute value) between the first three SC and rNC gradients. h) The variance explained of each gradient (principal component) for each SC and rNC matrix.

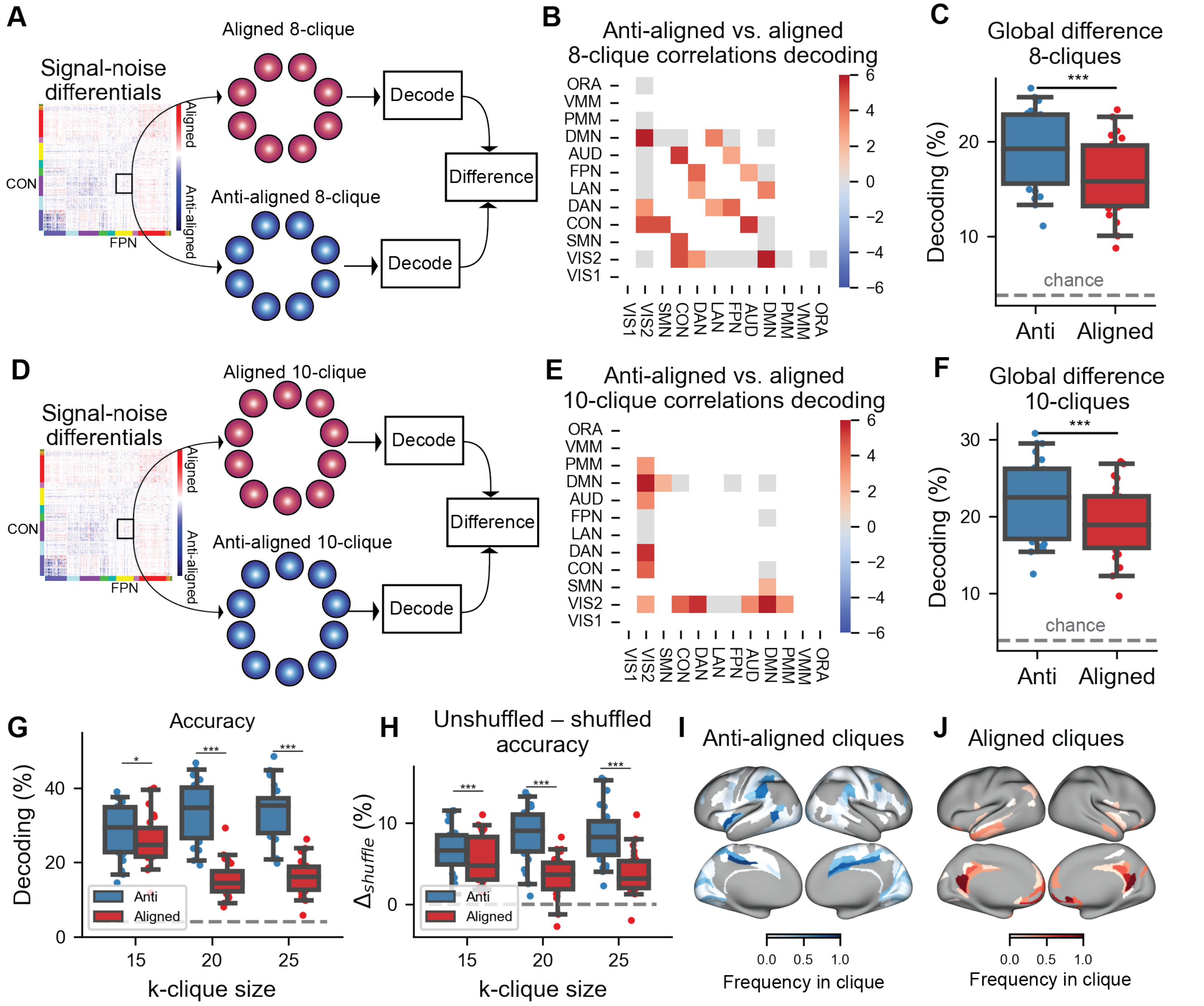

Supplementary Figure 5. Decoding analyses for different k-clique sizes – supplementary analyses for Fig. 6. a) Identifying 8-cliques within every pair of networks. b) Decoding accuracies for anti-aligned versus aligned 8-cliques for every pair of networks. Note that gray matrix elements indicate non-significant differences, and white elements indicate non-testable network configurations (due to non-existence of anti-aligned and/or aligned cliques of that size). c) Anti-aligned versus aligned decoding accuracies, averaged across all available network pairs. d-f) Same as a-c, but using 10-cliques. g) Decoding accuracies for 15, 20 (in the main text), and 25 anti-aligned and aligned cliques identified across the entire cortex. Anti-aligned cliques consistently had greater decoding accuracies than aligned cliques. h) The difference between unshuffled and shuffled decoding accuracies for anti-aligned and aligned cliques. Removing NCs impacted anti-aligned cliques significantly more than aligned cliques. i) We identified all possible 20-cliques for anti-aligned and j) aligned NCs, and plotted the frequency with which each region appeared in all cliques. Anti-aligned cliques tended to reside in sensory and motor areas, awhile aligned cliques were most frequently observed in medial prefrontal and posterior cingulate areas. (*** indicates $p<0.0001$; ** indicates $p<0.001$; * indicates $p<0.05$)
